# Characteristics of Avian Coronaviruses in China From 2020 to 2023

**DOI:** 10.1155/tbed/8919572

**Published:** 2026-04-10

**Authors:** Suchun Wang, Jinyu Sui, Junhui Pan, Shimeng Wei, Qian Qi, Chao Li, Xiyue Zhang, Kaicheng Wang

**Affiliations:** ^1^ China Animal Health and Epidemiology Center, Qingdao, 266032, China; ^2^ Key Laboratory of Animal Biosafety Risk Prevention and Control (South), Ministry of Agriculture and Rural Affairs, Qingdao, 266032, China, agri.gov.cn; ^3^ Key Laboratory of Animal Biosafety Risk Prevention, Qingdao, 266032, China

**Keywords:** avian coronavirus, cross-host transmission, distribution, epidemiological characteristics

## Abstract

Avian coronaviruses (CoVs) represent a significant threat to the poultry industry. To understand the epidemiological characteristics of avian CoVs in China, a nationwide surveillance was performed across 16 provinces from 2020 to 2023. More than 46,000 oropharyngeal/cloacal swab samples were collected and detected by RT‐PCR using specific primers for the RdRp gene. A total of 7370 Gammacoronaviruses (γCoVs) and 39 Deltacoronaviruses (δCoVs) were detected. The individual positive rates of γCoVs and δCoVs were 15.71% and 0.08%, respectively. The detected γCoV included 5825 infectious bronchitis viruses (IBVs), 1023 duck CoVs (DuCoVs), 511 pigeon CoVs (PiCoVs), and 11 goose CoVs (GoCoVs). While the γCoVs detected in this study demonstrated host specificity, some isolates indicated the potential for cross‐host transmission. The samples analyzed in this study were collected from 744 populations, including 191 poultry farms, 151 wholesale markets, 354 live poultry markets (LPMs), 38 slaughterhouses, 9 backyard flocks, and 1 wild bird observation point. Notably, wholesale markets (87.88%–95.96%) and LPMs (65.98%–95.77%) showed markedly high population positive rates. All avian CoV species detected in this study were identified in both wholesale markets and LPMs. Furthermore, concurrent circulation of two or more avian CoV species was detected in 48.10% (139/289) of positive LPMs and in 51.43% (72/140) of positive wholesale markets. The results indicate that LPMs and wholesale markets are key populations for the cross‐species transmission and genetic recombination of avian CoVs. Subsequently, a total of 3065 representative IBV samples were selected for phylogenetic analysis based on the S1 gene. Four genotypes, including GI (1688 strains), GIII (4 strains), GVI (418 strains), and GVII (48 strains), were identified. Among these, the GI‐19 (40.64%) and GVI‐1 (19.37%) were the prevalent lineages circulating in the Chinese poultry population. This study reveals the epidemiology, distribution, genetic, and phylogenetic characteristics of avian CoVs in China, providing important data for the research on the biological characteristics and cross‐host transmission mechanisms of avian CoVs.

## 1. Introduction

Coronaviruses (CoVs) are a group of RNA viruses that are widespread in nature. They mainly cause intestinal and/or respiratory symptoms in a variety of animals, such as pigs, cattle, horses, poultry, dogs, and bats as well as in humans. CoV pose a significant threat to global public health and animal husbandry due to their potential to cause severe disease. CoV belongs to the family Coronaviridae, and the subfamily Orthocoronavirinae, which includes four genera: Alphacoronavirus (αCoV), Betacoronavirus (βCoV), Gammacoronavirus (γCoV), and Deltacoronavirus (δCoV). Among these genera, αCoV and βCoV mainly infect humans and other mammals, while γCoV and δCoV mainly infect birds and pigs. The CoVs infecting domestic poultry mainly belong to γCoV, including avian infectious bronchitis virus (IBV), turkey CoV (TCoV), duck CoV (DuCoV), goose CoV (GoCoV), and pigeon CoV (PiCoV). CoVs found in wild birds are mainly classified as δCoV.

IBV was first discovered in 1930. It is the most extensively studied avian CoV and is now distributed worldwide. IBV is a highly contagious pathogen which mainly infects chickens but also can spread to other kinds of poultry species, causing severe losses to the poultry industry [1]. During viral replication, the IBV genome is easy to mutate and recombine, leading to the emergence of novel genotypes, lineages, or variants. Based on the molecular phylogenetic analysis of the S1 gene, IBVs are classified into 9 genotypes (GI‐GⅨ). Among these, GI genotype is globally prevalent and further subdivided into 30 lineages (GI‐1–GI‐30). GII, first characterized in Europe, has subsequently spread widely across Europe and South America. GIII and GV are prevalent in Australia and rarely reported in Asia. GIV, GVIII, and GIX all originated from North America; GVIII and GIX were first identified in Mexico in 2022 [2]. GIV is now established in Asia, while GVII has been detected in both Europe and China, and GVI‐1 appears limited to Asia [3]. New genotypes and lineages of IBV have been discovered continuously [3–7].

Besides IBV, poultry can also be infected by other kinds of CoVs, including TCoV, DuCoV, GoCoV, and PiCoV. TCoV was first identified in the United States in 1973, inducing acute severe enteric disease in turkeys [8]. To date, it remains endemic in most turkey farming regions around the world and continues to pose a significant economic loss despite the availability of various vaccines [1, 9, 10]. In China, the limited scale of turkey farming reduces the overall impact of TCoV. At present, surveillance data and epidemiological investigations on TCoV have not been systematically reported in China. DuCoV, which can cause severe diarrhea in ducks, was first discovered by next‐generation sequencing (NGS) in 2012. Regional surveillance in parts of China has revealed the individual infection rate of DuCoV as high as 4.38% in duck populations. Internationally, DuCoV infections have been documented in Norway, Sweden, and Laos. GoCoV and PiCoV were first detected in Norway in 2005 [11]. In 2019, Papineau et al. [12] conducted the whole‐genome analysis of CoV detected in geese from Canada and classified it into γCoV. Until now, PiCoVs have been detected in several countries, but the pathogenicity and pathogenic mechanisms of PiCoVs remain unclear and require further research. Several avian CoVs isolated from wild birds belong to δCoV, some of which exhibit host specificity in particular bird species and can spread across countries or regions through avian migration [13].

Avian CoVs exhibit genetic diversity and host variability, with distinct host tropism among different CoV species. Currently, there are no systematic nationwide surveillance reports for avian CoVs in China. To comprehensively investigate the distribution and epidemiological characteristics of different kinds of avian CoVs in Chinese poultry populations, a large‐scale surveillance was performed across 16 provinces in China from 2020 to 2023. The results provided important references for developing effective prevention and control strategies against avian CoV infections.

## 2. Material and Methods

### 2.1. Ethics Statement

This study was performed according to the animal welfare guidelines of the World Organization for Animal Health [14]. Swab samples were all collected with permission given by multiple relevant parties, including the Ministry of Agriculture of China, the China Animal Health and Epidemiology Center, and the relevant veterinary sections in the provincial and county government.

### 2.2. Sample Collection and Treatment

From 2020 to 2023, a total of 46,904 swab samples were collected from the 16 provinces of China. These samples were obtained as smears from both the cloacal and oropharyngeal tracts of each poultry. All 46,904 samples were clarified by centrifugation at 10,000 rpm for 5 min. Subsequently, 0.2 mL of the supernatant from each sample was inoculated into 10‐day‐old specific‐pathogen‐free (SPF) embryonated chicken eggs (Shandong Healthtec Laboratory Animal Breeding Company, Jinan, China) via the allantoic sac route. The inoculated eggs were incubated at 37°C for 3 days. Following incubation, allantoic fluid was collected from each inoculated egg and centrifuged at 12,000 rpm at 4°C for 10 min. Genomic RNA was extracted from supernatants using the QIAxtractor platform with the cador Pathogen 96 QIAcube HT kit (Qiagen, Hilden, Germany) according to the manufacturer’s instructions. All extracted RNA samples were stored at −80°C.

### 2.3. Distribution of Samples

The 46,904 swab samples were collected from 744 populations, including 191 poultry farms, 151 wholesale markets, 354 live poultry markets (LPMs), 38 slaughterhouses, nine backyard flocks, and one wild bird observation point. The samples were collected from 28,504 chickens, 12,613 ducks, 3019 pigeons, 2720 geese, 20 wild birds, 18 wild geese, and 10 quails (Table 1).

**Table 1:** The number of samples collected in the different hosts and population types.

Host	Poultry farm	Wholesale market	LPM	Slaughterhouse	Backyard flocks	Wild bird observation point	Total
Chicken	4357	12,122	9945	2022	58	—	28,504
Duck	1637	6235	3676	992	73	—	12,613
Pigeon	35	846	2111	27	—	—	3019
Geese	1105	1024	449	115	27	—	2720
Wild bird	—	—	—	—	—	20	20
Wild geese	—	—	18	—	—	—	18
Quail	—	5	5	—	—	—	10
Total	7134	20,232	16,204	3156	158	20	46,904

### 2.4. Detection and Analysis of Avian CoV

To identify the species of avian CoV infecting Chinese poultry, the RdRp gene was amplified by RT‐PCR using extracted RNA. The RT‐PCR method can detect all the animal CoVs [15]. The amplified product was about 600 bp in length. The amplified products were sequenced, and the obtained sequences were compared with 51 reference sequences using MEGA7.0 software for analysis. Information on the 51 reference sequences is listed in Table S1. A phylogenetic tree was constructed using the neighbor‐joining method in MEGA7.0 software to classify the detected animal CoVs by species [16]. The RT‐PCR forward primer was CoV‐F 5′‐GGTTGGGATTAY(C/T)CCW(A/T) AAR(A/G)TGY(CT)GA‐3′, and the reverse primer was CoV‐R 5′‐Y(C/T)TGTGAACAAAAY(C/T)TCR(A/G)TG W(A/T)GGACC‐3′. The RdRp gene was amplified using the Evo M‐MLV One‐Step RT‐PCR Kit (Accurate Biotechnology (Hunan) Co., Ltd., Hunan, China; AG11606). RT‐PCR was performed in a 25 μL reaction system containing 12.5 μL of 2× One‐step Master Mix, 1 μL of Evo M‐MLV RTase, 1 μL of each primer (10 μM), 6.5 μL of RNase Free dH_2_O, and 3 μL of template RNA. The reaction was initially incubated at 50°C for 30 min, followed by the denaturation at 94°C for 2 min, and then 30 cycles of 94°C for 30 s, 55°C for 30 s, 72°C for 45 s, with a final extension step at 72°C for 5 min.

The epidemiological distribution characteristics of avian CoVs in Chinese poultry populations were analyzed based on the detection results, including their temporal, geographical, and host‐related distributions.

### 2.5. Genotype and Lineages Analysis of IBVs

A total of 3065 representative IBV positive samples were selected for S1 gene amplification. The criterion for selecting representative IBV‐positive samples is as follows: First, all IBV‐positive samples are screened based on the results of RT‐PCR tests. Then, one to three IBV samples are randomly selected from different types of animals in each field site that had positive samples. The S1 genes were amplified with the Evo M‐MLV One‐Step RT‐PCR Kit (Accurate Biotechnology (Hunan) Co., Ltd., Hunan, China; AG11606) using primers IB‐S1‐F 5′‐AAAACGCTGCACGCAAATTA‐3′ (forward primer) and IB‐S1‐R 5′‐AATAAAACCTTGACCTACTCTACCA‐3′ (reverse primer). The specifically amplified product was ~1600 bp in length. The RT‐PCR reaction was initially incubated at 50°C for 30 min, followed by the denaturation at 94°C for 2 min, and then 30 cycles of 94°C for 30 s, 55°C for 30 s, 72°C for 2 min, with a final extension step at 72°C for 5 min. A phylogenetic tree was constructed by the neighbor‐joining method using the MEGA7.0 software for genetic typing analysis based on the amplified S1 gene sequences [16]. The reference sequences used for the phylogenetic tree of the IBV S1 gene are listed in Table S2. An analysis was performed on the prevalence and distribution of various IBV genotypes and lineages in poultry populations in China.

## 3. Results

### 3.1. Temporal Distribution of Avian CoV

**Figure 1:**
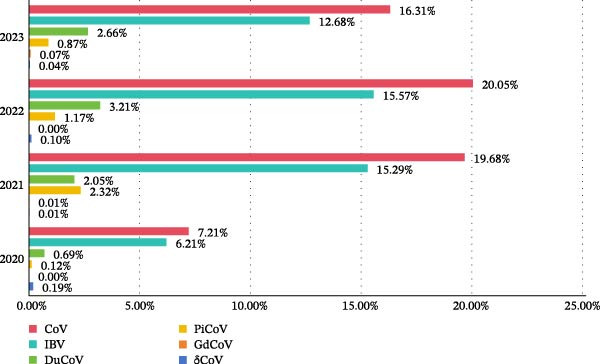
The individual positive rates of avian coronaviruses from 2020 to 2023.

A total of 7370 γCoV and 39 δCoV were identified from the 46,904 clinical samples by RT‐PCR amplification and sequence analysis of RdRp genes. The individual positive rate of avian CoVs in the poultry was 15.79%. Among the γCoV, 5,825 IBVs, 1023 DuCoVs, 511 PiCoVs, and 11 GoCoVs were identified, with individual positive rates of 12.42%, 2.18%, 1.09%, and 0.02%, respectively. The individual positive rates of avian CoVs and the five virus species in each year were shown in Figure 1. Overall, the individual positive rate for avian CoVs showed an overall upward trend from 2020 to 2022. In 2023, it decreased slightly while remaining stable, but still stayed at a relatively high level. The IBV individual positive rate was consistently highest every year, followed by DuCoV in most of the years (2020, 2022, and 2023).

### 3.2. Geographical Distribution of Avian CoV

IBV is widely distributed among the poultry populations in China and has been detected in all 16 provinces. Among them, the individual positive rate (26.53%) in Shandong province is the highest, followed by Hunan (21.01%), Jiangsu (20.42%), Anhui (20.39%), Hubei (20.01%), Sichuan (18.42%), Guangdong (18.10%), and Henan (17.69%), all of which are higher than the total individual positive rate. Additionally, Guangdong and Guangxi provinces had the highest number of detected avian CoV species. All five species of avian CoV, including IBV, DuCoV, PiCoV, GoCoV, and δCoV, were detected in these two provinces. The individual positive rate of different avian CoV species in each province is shown in Table 2.

**Table 2:** Number of samples and individual positive rates of avian coronaviruses in each province.

Province	Number of samples	Number of avian coronavirus positive samples (individual positive rate)					
		IBV	DuCoV	PiCoV	GoCoV	δCoV	Total
Anhui	3840	557 (14.51%)	128 (3.33%)	98 (2.55%)	0	0	783 (20.39%)
Fujian	3830	418 (10.91%)	66 (1.72%)	71 (1.85%)	0	0	555 (14.45%)
Guangdong	4440	487 (10.97%)	168 (3.78%)	28 (0.63%)	8 (0.18%)	4 (0.09%)	695 (18.10%)
Guangxi	3809	264 (6.93%)	129 (3.39%)	47 (1.23%)	1 (0.10%)	6 (0.16%)	447 (11.74%)
Guizhou	960	107 (11.15%)	13 (1.35%)	12 (1.25%)	1 (0.10%)	0	133 (13.85%)
Henan	3832	653 (17.04%)	3 (0.08%)	22 (0.57%)	0	0	678 (17.69%)
Heilongjiang	1913	57 (2.98%)	0	0	0	0	57 (2.98%)
Hubei	3689	584 (15.83%)	144 (3.90%)	10 (0.27%)	0	0	738 (20.01%)
Hunan	1918	242 (12.62%)	149 (7.77%)	12 (0.63%)	0	0	403 (21.01%)
Jiangsu	3840	685 (17.84%)	10 (0.26%)	89 (2.32%)	0	0	784 (20.42%)
Jiangxi	3990	446 (11.18%)	63 (1.58%)	87 (2.18%)	0	0	596 (14.94%)
Ningxia	2705	200 (7.39%)	2 (0.07%)	2 (0.07%)	0	0	204 (7.54%)
Shandong	720	189 (26.25%)	2 (0.28%)	0	0	0	191 (26.53%)
Shanghai	300	12 (4.00%)	0	0	0	0	12 (4.00%)
Sichuan	3338	443 (13.27%)	129 (3.86%)	14 (0.42%)	0	29 (0.87%)	615 (18.42%)
Yunnan	3780	481 (12.72%)	17 (0.45%)	19 (0.50%)	1 (0.03%)	0	518 (13.70%)
Total	46,904	5831 (12.42%)	1023 (2.18%)	511 (1.09%)	11 (0.02%)	39 (0.08%)	7409 (15.79%)

### 3.3. Host Distribution of Avian CoV

**Figure 2:**
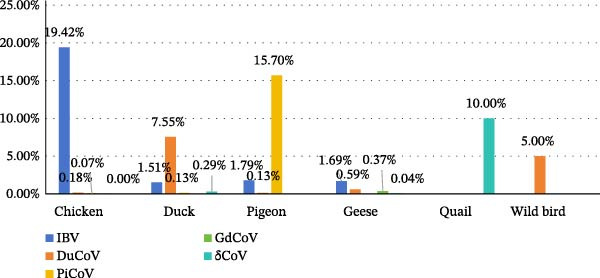
Individual positive rates of various avian coronaviruses in different hosts.

Avian CoVs were detected in various hosts, including chickens, ducks, pigeons, geese, wild birds, and quails, but not in wild geese. The individual positive rate of avian CoVs was the highest in chickens (19.67%), followed by pigeons (17.62%) and ducks (9.47%), while the individual positive rate in geese was lower (2.68%). The individual positive rate of various avian CoVs in different hosts was shown in Figure 2. The results indicated that chickens, ducks, and pigeons remain the primary hosts for avian CoV infections. One sample collected from wild birds tested positive for DuCoV, and one sample collected from quails tested positive for δCoV, suggesting the potential risk of cross‐host transmission. Epidemiological surveillance of avian CoVs in poultry populations can provide a comprehensive understanding of their prevalence and trends in China, offering valuable insights for the prevention and control of avian CoV infections.

Analysis of avian CoVs detected in different hosts revealed host specificity among different species of avian CoVs. Specifically, 98.72% (5535/5607) of the avian CoVs detected in chickens were IBVs. In total of 79.67% (952/1195) of avian CoVs detected in ducks were DuCoVs. In total of 89.10% (474/532) of avian CoVs detected in pigeons were PiCoVs. Among the avian CoVs detected in geese, IBVs were the predominant species (63%), while DuCoVs and GoCoVs represented 21% and 13.7%, respectively (Table 3). Furthermore, IBVs, DuCoVs, and PiCoVs demonstrated the ability to infect multiple hosts, indicating host diversity and the potential risk of cross‐host transmission.

**Table 3:** The number and percentage of the avian coronavirus species in different hosts.

Host	Number of samples	IBV (%)	DuCoV (%)	PiCoV (%)	GoCoV (%)	δCoV (%)	Number of avian coronavirus positive sample in total
Chicken	28,504	**5535 (98.72%)**	50 (0.89%)	20 (0.36%)	1 (0.02%)	1 (0.02%)	5607
Duck	12,613	190 (15.90%)	**952 (79.67%)**	17 (1.42%)	0	36 (3.01%)	1195
Pigeon	3019	54 (10.15%)	4 (0.75%)	**474 (89.10%**)	0	0	532
Geese	2720	46 (63.01%)	16 (21.92%)	0	10 (13.70%)	1 (1.37%)	73
Quail	10	0	0	0	0	1 (100%)	1
Wild geese	18	0	0	0	0	0	0
Wild bird	20	0	1 (100%)	0	0	0	1
Total	46,904	5825 (78.62%)	1023 (13.81%)	511 (6.90%)	11 (0.15%)	39 (0.53%)	7409

*Note:* The bold values indicate that this avian coronavirus species accounts for the highest proportion of all detected avian coronaviruses in this animal species.

### 3.4. Population Distribution of Avian CoVs

**Figure 3:**
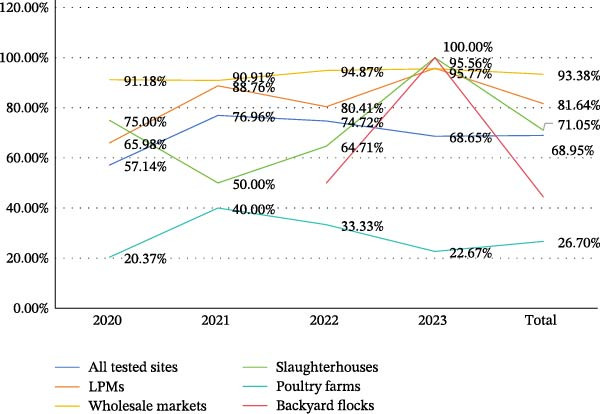
Population positive rates of avian coronaviruses in different population types from 2020 to 2023.

Among all the detected population types, the population positive rate of avian CoVs is consistently higher in LPMs and wholesale markets. All avian CoV species detected in this study were found in both of these two types of population (Table S3). Furthermore, two or more avian CoV species were detected in 48.10% (139/289) of positive LPMs and in 51.43% (72/140) of positive wholesale markets (Table S4). The population positive rate in slaughterhouses ranged between 50.00% and 100% (Table 4). The population positive rate in poultry farms was persistently low, which may be attributable to their remote locations, enclosed environments, standardized vaccination protocols, and proper farming management practices. Data from backyard flocks and wild bird observations were less reliable due to the limited sample sizes. The population positive rate of avian CoVs in different population types from 2020 to 2023 was shown in Figure 3.

**Table 4:** The population positive rates of avian coronaviruses in different types of populations.

Time	The population positive rate of avian coronavirus in different population types (number of positive populations/number of populations)						
	Live poultry markets	Wholesale markets	Poultry farmers	Slaughterhouse	Backyard flocks	Wild bird observation point	Total
2020	65.98% (64/97)	91.18% (31/34)	0 (0/3)	75.00% (6/8)	20.37% (11/54)	—	57.14% (112/196)
2021	88.76% (79/89)	87.88% (29/33)	0 (0/1)	50.00% (3/6)	40.00% (14/35)	100.00% (1/1)	76.36% (126/165)
2022	80.41% (78/97)	94.87% (37/39)	50.00% (1/2)	64.71% (11/17)	33.33% (9/27)	—	74.72% (136/182)
2023	95.77% (68/71)	95.96% (43/45)	100.00% (3/3)	100.00% (7/7)	22.67% (17/75)	—	68.65% (138/201)
Total	81.64% (289/354)	93.38% (141/151)	44.44% (4/9)	71.05% (27/38)	26.70% (51/191)	100.00% (1/1)	68.95% (513/744)

### 3.5. Genotype Analysis of IBVs

Out of the 5825 IBV positive samples, 3065 representative samples were selected for S1 gene amplification, sequencing, and phylogenetic analysis. The results showed that four genotypes were detected among the 3065 samples, including GI (1,688), GIII (4), GVI (418), and GVII (48), as well as the emerging recombinant strains discovered in recent years (907). Of the 1688 GI genotype IBVs, nine lineages were identified, including GI‐1, GI‐7, GI‐9, GI‐13, GI‐19, GI‐22, GI‐23, GI‐28, and GI‐29 (Figure S1).

**Figure 4:**
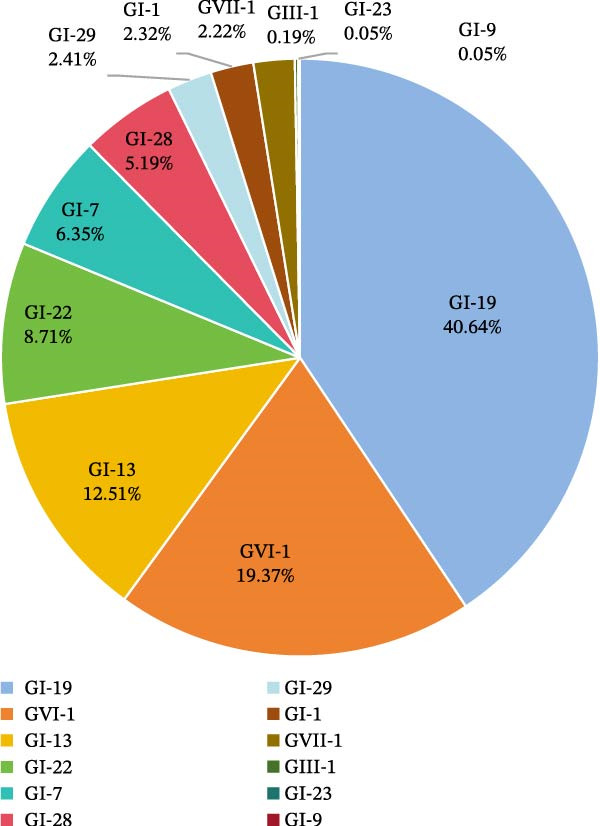
The percentage of 12 IBV lineages in IBV positive samples.

The GI‐19 (40.64%) and GVI‐1 (19.37%) lineages accounted for 60% (1295/2158) of IBV positive samples, excluding the emerging recombinant IBV positive samples (Figure 4). These were the most widely circulating lineages in the Chinese poultry. Previous studies have analyzed the emerging recombinant IBV identified during this surveillance, which originated from genetic recombination between GI‐19 or GVI‐1 backbone strains and the S gene of TCoVs or guinea fowl CoVs [17]. The number and percentage of IBV lineages in the 16 provinces are listed in Table 5. Among them, the GI‐13 lineage IBVs had the most extensive geographic distribution, being detected in all the 16 provinces. Both GI‐19 and GVI‐1 lineage IBVs were identified in 15 provinces. The GI‐19 lineage was predominant in the Jiangsu and Henan provinces, while the GVI‐1 lineage was predominant in the Hubei province.

**Table 5:** The number and percentage of IBV lineages in the 16 provinces.

Province	GI‐1	GI‐7	GI‐9	GI‐13	GI‐19	GI‐22	GI‐23	GI‐28	GI‐29	GIII‐1	GVI‐1	GVII‐1
Anhui	2 (1.18%)	6 (3.53%)	—	10 (5.88%)	64 (37.65%)	7 (4.12%)	1 (0.59%)	14 (8.24%)	8 (4.71%)	1 (0.59%)	50 (29.41%)	7 (4.12%)
Fujian	—	18 (10.78%)	—	45 (26.95%)	39 (23.35%)	20 (11.98%)	—	—	3 (1.80%)	—	41 (24.55%)	1 (0.60%)
Guangdong	3 (2.19%)	4 (2.92%)	—	33 (24.09%)	32 (23.36%)	20 (14.60%)	—	5 (3.65%)	6 (4.38%)	—	26 (18.98%)	8 (5.84%)
Guangxi	3 (3.80%)	6 (7.59%)	—	14 (17.72%)	25 (31.65%)	2 (2.53%)	—	7 (8.86%)	8 (10.13%)	—	8 (10.13%)	6 (7.59%)
Guizhou	1 (4.35%)	6 (26.09%)	—	6 (26.09%)	2 (8.70%)	—	—	1 (4.35%)	1 (4.35%)	—	3 (13.04%)	3 (13.04%)
Henan	4 (1.37%)	11 (3.75%)	—	20 (6.83%)	185 (63.14%)	48 (16.38%)	—	3 (1.02%)	1 (0.34%)	—	20 (6.83%)	1 (0.34%)
Heilongjiang	15 (35.71%)	—	—	1 (2.38%)	16 (38.10%)	10 (23.81%)	—	—	—	—	—	—
Hubei	1 (0.49%)	25 (12.32%)	—	28 (13.79%)	30 (14.78%)	2 (0.99%)	—	12 (5.91%)	—	1 (0.49%)	104 (51.23%)	—
Hunan	2 (3.17%)	5 (7.94%)	—	4 (6.35%)	22 (34.92%)	—	—	18 (28.57%)	5 (7.94%)	—	7 (11.11%)	—
Jiangsu	—	2 (0.63%)	—	38 (11.91%)	201 (63.01%)	34 (10.66%)	—	7 (2.19%)	2 (0.63%)	—	35 (10.97%)	—
Jiangxi	9 (6.16%)	2 (1.37%)	—	18 (12.33%)	40 (27.40%)	24 (16.44%)	—	3 (2.05%)	3 (2.05%)	2 (1.37%)	44 (30.14%)	1 (0.68%)
Ningxia	2 (2.35%)	3 (3.53%)	—	15 (17.65%)	38 (44.71%)	12 (14.12%)	—	—	1 (1.18%)	—	12 (14.12%)	2 (2.35%)
Shandong	1 (0.76%)	—	—	10 (7.63%)	47 (35.88%)	—	—	40 (30.53%)	—	—	33 (25.19%)	—
Shanghai	—	2 (20.00%)	—	3 (30.00%)	—	3 (30.00%)	—	—	—	—	2 (20.00%)	—
Sichuan	5 (3.68%)	19 (13.97%)	1 (0.74%)	19 (13.97%)	54 (39.71%)	4 (2.94%)	—	2 (1.47%)	6 (4.41%)	—	14 (10.29%)	12 (8.82%)
Yunnan	2 (1.30%)	28 (18.18%)	—	6 (3.90%)	82 (53.25%)	2 (1.30%)	—	—	8 (5.19%)	—	19 (12.34%)	7 (4.55%)
Total	50 (2.32%)	137 (6.35%)	1 (0.05%)	270 (12.51%)	877 (40.64%)	188 (8.71%)	1 (0.05%)	112 (5.19%)	52 (2.41%)	4 (0.19%)	418 (19.37%)	48 (2.22%)

## 4. Discussion

Since the initial identification of avian CoVs, these viruses have caused significant economic losses to the poultry industries worldwide. The emergence of highly pathogenic human CoVs, such as SARS‐CoV in 2003, MERS‐CoV in 2012, and COVID‐19 in 2019, has substantially increased global attention to CoVs and stimulated renewed research interest in avian CoVs. Among avian CoV, IBV is the most extensively studied, but other avian CoVs are also evolving rapidly. Due to the high mutation frequency characteristic of CoVs, novel strains and variants are continually being identified, necessitating further investigation into their pathogenicity, transmission mechanisms, and epidemiological patterns. Therefore, systematic surveillance of the distribution of CoVs in avian populations is a critical priority for essential public and animal health.

This nationwide study of avian CoVs was conducted from 2020 to 2023. A total of 46,904 clinical samples were collected from 744 populations across 16 provinces in China. A total of 7409 avian CoVs were detected, with an individual positive rate of 15.79% and a population positive rate of 68.95%. The individual positive rate of avian CoVs was the highest in chickens (19.67%), followed by pigeons (17.62%) and ducks (9.47%). In 2024, Wang et al. [18] conducted a nationwide investigation of avian CoV. The results showed that the individual positive rate of ACoVs was 20.60% and the positive rate at the site reached up to 56.21%, indicating a broad contamination of the ACoVs. This is consistent with the results of this study, indicating a wide distribution of avian CoVs in poultry populations, along with consistently high positive rates observed at both the individual and population levels.

Avian CoVs exhibit significant genetic diversity and host specificity. Wild birds can be infected with both γCoV and δCoV simultaneously, playing a crucial role in the transmission of avian CoV [17, 19–21]. In poultry, the emergence and spread of novel avian CoVs are primarily driven by either cross‐host transmission from wild birds followed by adaptive evolution within the new hosts or genetic recombination among different avian CoV species. Chu et al. [22] conducted surveillance and analysis of CoVs in wild birds in Hong Kong and Cambodia. The results showed that frequent intergenus and interspecies transmissions of γCoVs among the monitored bird species, whereas δCoVs exhibited more stringent host specificity, which is consistent with the results of the present study. Since the detection method used in this study was based on viral RNA detection, and viral isolation and whole‐genome characterization were not performed for all viruses, we can only conclude that some γCoV isolates indicate the potential for cross‐host transmission. Guo et al. [23] considered that the global dissemination of γCoVs and δCoVs among wild bird populations and other animal groups is likely facilitated by multiple migratory pathways, which interconnect bird communities across different countries and regions. Therefore, simultaneous surveillance of CoVs in both domestic poultry and wild birds will improve our understanding of the cross‐regional and cross‐host transmission mechanisms of avian CoVs.

IBV is the most widely distributed and currently poses the greatest threat to the Chinese poultry industry. Although chickens are the primary hosts of IBV, the virus has also been detected in various domestic poultry species, demonstrating the continuous expansion of its host range. The high mutation frequency of IBV, driven by the error‐prone nature of RNA‐dependent RNA polymerase and frequent recombination events during replication, leads to the emergence of novel strains, lineages, or genotypes. In the surveillance, four genotypes comprising 12 lineages were identified in Chinese poultry populations, with the GI‐19 and GVI‐1 lineages currently dominant. The GI‐19 lineage was first identified in 1990s in China [24] and has been reported in multiple countries and regions, including Asia [25–27], Europe [28–31], Africa [32] and the Middle East [33]. Since the initial detection of GVI genotype in China in 2007, it has become prevalent throughout East Asia [34]. Data analysis in this study indicated that the positive rate of GVI genotype remains relatively high in Chinese poultry populations. However, tests conducted by Chen et al. [35] on clinical samples between January 2021 and June 2022 showed that lineages GI‐19 and GI‐7 have become the most prevalent IBV strains in China. The dominant lineages may differ with sample source, but the GI‐19 lineage consistently predominates. A study conducted by Rafique et al. [3] in Pakistan (2018) reported that the GI‐1 and GI‐13 lineages accounted for 43% and 51% of the detected IBV strains, respectively. However, Saleem et al. [36] reported that GI‐24 has emerged as the predominant circulating lineage during 2017–2020 in Pakistan, a profile distinct from that of the lineages circulating in China. However, since live‐attenuated IBV vaccines are widely used in many regions of China, and the detection method employed in this study cannot distinguish between vaccine strains and wild‐type strains, vaccination effects should be comprehensively taken into consideration when interpreting the positive rates of viral lineages such as GI‐1 and GI‐19.

Vaccination is a key strategy for controlling avian CoV infections. At present, the existing vaccines against avian CoVs only target IBVs and TCoVs. In China, only vaccines against IBVs are available. It is unknown whether these vaccines can provide cross‐protection against other avian CoV species. The genotype and lineage of IBVs are numerous, and the cross‐protective immunity varies significantly between different genotypes, lineages, and strains. Furthermore, the distinct geographical distribution patterns of certain lineages substantially complicate vaccine selection and implementation strategies.

LPMs and wholesale markets have long served as major reservoirs for avian pathogens, facilitating cross‐host transmission and genetic recombination. Consistent with the findings of Zhuang et al. [37, 38], this study reveals persistently high individual positive rates for avian CoVs in LPMs and wholesale markets, likely due to the concentration of avian species in these populations [23]. Additionally, a greater variety of avian CoV species were detected in these two population types. Even in individual populations, four species of avian CoVs were detected. The results suggested that LPMs and wholesale markets may facilitate mixed infection and genetic recombination of avian CoVs. These conditions significantly increase the likelihood of pathogen mutation and the emergence of novel variants, thereby promoting cross‐host transmission and posing a significant risk to public health security.

## 5. Conclusion

γCoVs are generally host‐specific; however, some isolates indicated the potential for cross‐host transmission. IBV and DuCoV are widespread in Chinese poultry populations. IBV demonstrates the most extensive geographic distribution and the greatest degree of genotypic diversity, as well as complex epidemiological patterns. This study identified four genotypes, including GI, GIII, GVI, and GVII, circulating in China. Among these, GI‐19 and GVI‐1 were the predominant lineages in the poultry population. LPMs and wholesale markets exhibited markedly high population positive rates, with a variety of avian CoV species being detected. These may serve as critical populations for the reservoir, genetic variation, and cross‐host transmission of avian CoVs. Strengthening epidemiological surveillance, developing vaccines, researching cross‐host transmission mechanisms, and assessing the risks of avian CoVs can provide critical data to support disease prevention and control, while also providing valuable references for the study of other CoVs.

## Author Contributions

Kaicheng Wang contributed to the conceptualization. Kaicheng Wang and Suchun Wang contributed to the analysis and interpretation of data. Junhui Pan, Shimeng Wei, Qian Qi, Chao Li, and Xiyue Zhang contributed to the resources. Kaicheng Wang, Suchun Wang, Jinyu Sui, Junhui Pan, Shimeng Wei, Qian Qi, and Chao Li contributed to the investigation. Kaicheng Wang, Suchun Wang, and Jinyu Sui drafted the manuscript.

## Funding

This work was supported by the National Key Research and Development Program of China (Grant 2023YFD1800604).

## Disclosure

All authors reviewed the manuscript and approved the final version of the manuscript for publication.

## Conflicts of Interest

The authors declare no conflicts of interest.

## Supporting Information

Additional supporting information can be found online in the Supporting Information section.

## Supporting information


**Supporting Information** Table S1: Reference sequences used for the phylogenetic analysis of animal coronavirus RdRp gene. Table S2: Lists the reference sequences employed in the phylogenetic analysis of the IBV S1 gene. Table S3: Lists the number of positive samples for different avian coronavirus species that were detected in different population types. Table S4: The number of positive populations for each detected avian coronavirus species. Figure S1: The phylogenetic tree constructed from the IBV positive samples.

## Data Availability

The data that support the findings of this study are available from the corresponding author upon reasonable request.
