# Supplementary material for: Characteristics of Avian Coronaviruses in China From 2020 to 2023
Source: Transbound Emerg Dis. 2026 Apr 10;2026:8919572. doi: 10.1155/tbed/8919572 (PMC13067302; doi:10.1155/tbed/8919572)
Supplement: Supplementary file 1 — Supporting Information Table S1: Reference sequences used for the phylogenetic analysis of animal coronavirus RdRp gene. Table S2: Lists the reference sequences employed in the phylogenetic analysis of the IBV S1 gene. Table S3: Lists the number of positive samples for different avian coronavirus species that were detected in different population types. Table S4: The number of positive populations for each detected avian coronavirus species. Figure S1: The phylogenetic tree constructed from the IBV positive samples. [file TBED-2026-8919572-s001.docx]

Table S1 Reference sequences for the phylogenetic tree of animal coronavirus RdRp gene

| Accession | Species | Isolate |
| --- | --- | --- |
| DQ011855 | Porcine hemagglutinating encephalomyelitis virus | VW572 |
| DQ084199 | bat SARS coronavirus | HKU3-2 |
| DQ497008 | SARS coronavirus | MA-15 |
| DQ915164 | Bovine_coronavirus | Alpaca |
| EF424621 | Sable antelope coronavirus | US/OH1/2003 |
| EF424622 | Giraffe_coronavirus | US/OH3-TC/2006 |
| FJ376620 | Bulbul coronavirus | HKU11-796 |
| FJ425189 | Sambar_deer_coronavirus | US/OH-WD388/1994 |
| GU396675 | Western sandpiper coronavirus | KR28 |
| GU396677 | Brent_goose coronavirus | KR-69 |
| GU396678 | Brent_goose coronavirus | KR88 |
| HM245926 | Mink coronavirus | WD1133 |
| JF792616 | Rat coronavirus | 681 |
| JQ404410 | Canine_coronavirus | TN-449 |
| JX560761 | PEDV | SD-M |
| KF268338 | Murine_coronavirus | MHV/BHKR_lab/USA/icA59 L94P/2012 |
| KF793824 | Bottlenose_dolphin_coronavirus_HKU22 | CF090325 |
| KJ567050 | Porcine deltacoronavirus | 8734/USA-IA/2014 |
| KJ601777 | Deltacoronavirus | PDCoV/USA/Illinois133/2014 |
| KM027261 | MERS | Makkah C9355/KSA/Makkah/2014-04-15 |
| KM975741 | PEDV | USA/MO/2014/03293 |
| KP033137 | Goose-dominant Coronavirus | GdCoV/GS/guangdong/F38/2014 |
| KT368891 | Camel coronavirus HKU23 | camel/Riyadh/Ry123/2015 |
| KU182964 | Betacoronavirus | JTMC15 |
| KX900402 | TGEV | TGEV/Mex/145/2008 |
| KY432458 | Bat_coronavirus | 16BO133 |
| KY566210 | Feline_coronavirus | HLJ/HRB/2016/11 |
| KY862032 | Human coronavirus | NL63/FRAEPI/Caen/2005/03 |
| LC061274 | Equine coronavirus | Obihiro12-2 |
| LC364344 | Pigeon coronavirus | UAE-HKU29 271F |
| MG518518 | Water deer coronavirus | W17-18 |
| MG772934 | Bat SARS-like_coronavirus | baT-SL-CoVZXC21 |
| MH043954 | Bovine coronavirus | 4-17-08 |
| MK983498 | Infectious bronchitis virus | CdCoV/CK/Hubei/3-7-9/2018 |
| MK983503 | Infectious bronchitis virus | CdCoV/CK/Hainan/11-1-3/2018 |
| MK983513 | Duck-dominant coronavirus | PdCoV/PG/Jiangsu/9-1-3/2018 |
| MK983515 | Duck-dominant coronavirus | PdCoV/PG/Jiangsu/9-7-9/2018 |
| MK983516 | Pigeon-dominant Coronavirus | PdCoV/PG/Hebei/15-4-6/2018 |
| MK983518 | Pigeon-dominant Coronavirus | DdCoV/DK/Hunan/5-1-3/2018 |
| MZ490117 | Severe acute respiratory syndrome coronavirus | SARS-CoV-2/human/USA/UT-UPHL-2104411907/2021 |
| NC001846 | Mouse hepatitis virus | MHV-A59 C12 |
| NC003045 | Bovine coronavirus | BCoV-ENT |
| NC004718 | SARS coronavirus | Tor2 |
| NC009020 | Bat_coronavirus | HKU5 |
| NC009021 | Bat coronavirus | HKU9-1 |
| NC010646 | Beluga Whale coronavirus | SW1 |
| NC016994 | Night-heron coronavirus | HKU19 |
| NC017083 | Rabbit coronavirus | HKU14 |
| NC023760 | Mink_coronavirus | WD1127 |
| NC045512 | Severe acute respiratory syndrome coronavirus 2 | Wuhan-Hu-1 |
| NC046965 | Canada goose coronavirus | Cambridge Bay 2017 |

Table S2 Reference sequences for the phylogenetic tree of the IBV S1 gene

| Accession | Isolate | Genotype |
| --- | --- | --- |
| AY561711 | M41_USA | GI-1 |
| FJ888351 | H120_Netherlands | GI-1 |
| AF151959 | K87_New | GI-10 |
| AF151960 | T6_New | GI-10 |
| GU393339 | IBV/Brazil/351/1984 | GI-11 |
| JX182783 | UFMG/1141_Brazil | GI-11 |
| X15832 | D274_The_Netherlands | GI-12 |
| X52084 | D3896__Netherlands | GI-12 |
| EU914938 | Moroccan/G/83 | GI-13 |
| Z83975 | UK4/91 | GI-13 |
| FN182277 | NGA3242006 | GI-14 |
| X87238 | B1648_Belgium | GI-14 |
| FJ807932 | B4_Korea | GI-15 |
| FJ807944 | K620/02_Korea | GI-15 |
| AF286302 | Q1_China | GI-16 |
| KJ941019 | IZO_28/86_Italy | GI-16 |
| AF027509 | CV-56b_USA | GI-17 |
| AF510656 | AL/6609/98_USA | GI-17 |
| AY296744 | JP8127_Japan | GI-18 |
| KC577391 | 53XJ-99II_China | GI-18 |
| AF193423 | QXIBV_China | GI-19 |
| AY189157 | LX4_China | GI-19 |
| DQ070840 | SDW_China | GI-2 |
| GU393336 | Holte_USA | GI-2 |
| AF349620 | Qu_16_Canada | GI-20 |
| AF349621 | Qu_mv_Canada | GI-20 |
| AJ457137 | Italy/02 | GI-21 |
| DQ064808 | Spain/98/313 | GI-21 |
| DQ167147 | CK/CH/LSC/99I | GI-22 |
| GQ265940 | HN08_China | GI-22 |
| AF093796 | Israel_Var_II_ | GI-23 |
| KY805846 | Egyptian_Var_II | GI-23 |
| KF757447 | V13_India | GI-24 |
| KF809796 | IBV506_India | GI-24 |
| KM660636 | GA/10216/2010_USA | GI-25 |
| KP085595 | GA/12274/2012_USA | GI-25 |
| FN182268 | NGA/BP61/2007_Nigeria | GI-26 |
| FN182270 | NGA/N545/2006_Nigeria | GI-26 |
| GU301925 | Georgia_08_USA | GI-27 |
| KM660634 | GA/12341/2012_USA | GI-27 |
| JX291989 | GX-NN-13_Chin | GI-28 |
| KX640829 | ck/CH/LGX/111119 | GI-28 |
| KY407556 | 10114/14_China | GI-29 |
| KY407558 | 10118/14_China | GI-29 |
| L14069 | Gray_USA | GI-3 |
| L14070 | JMK_USA | GI-3 |
| AY251816 | GX2-98_China | GI-4 |
| L18988 | Holte_USA | GI-4 |
| DQ490215 | V2/02_Australia | GI-5 |
| U29519 | N1/62_Australia | GI-5 |
| DQ515802 | J9_China | GI-6 |
| U29519 | Vic_S_Australia | GI-6 |
| AY606320 | TP/64_Taiwan | GI-7 |
| DQ646405 | TW2575.98 | GI-7 |
| M99484 | SE_17_USA | GI-8 |
| Q964061 | L165_USA | GI-8 |
| AF006624 | Ark_DPI_USA | GI-9 |
| DQ912831 | CAL99_USA | GI-9 |
| M21968 | V1397_The_Netherlands | GII-1 |
| M21971 | D1466_The_Netherlands | GII-1 |
| JN176213 | N1/08_Australia | GIII-1 |
| U29450 | N1/88_Australia | GIII-1 |
| U29521 | V18/91_Australia | GIII-1 |
| AF274436 | AR/6386/97_USA | GIV-1 |
| EU283066 | GA/Avial1_S1_USA | GIV-1 |
| U77298 | DE/072/92_USA | GIV-1 |
| FJ235191 | V1/07_Australia | GV-1 |
| FJ235194 | N1/03_Australia | GV-1 |
| JX018208 | 018_Australia | GV-1 |
| GQ265948 | TC07-2_China | GVI-1 |
| JF804677 | K23/10_South_Korea | GVI-1 |
| KF007209 | SDIB781/2012_China | GVI-1 |
| KM365468 | GX-NN130021_China | GVII-1 |
| MH924835 | 10636/26_China | GVII-1 |
| AY789942 | PA/1220/98_USA | GVIII |
| MT591566 | CK/DE/IB80/2016_Germany | GVIII |

Table S3 Number of positive samples for different avian coronavirus species that were detected in different population types

| Population | IBV | DuCoV | PiCoV | GoCoV | δCoV |
| --- | --- | --- | --- | --- | --- |
| Live poultry markets | 2248 | 259 | 372 | 3 | 7 |
| Wholesale markets | 2792 | 635 | 137 | 8 | 32 |
| Poultry farmers | 288 | 10 | 2 | 0 | 0 |
| Slaughterhous | 487 | 118 | 0 | 0 | 0 |
| Backyard flocks | 10 | 0 | 0 | 0 | 0 |
| Wild bird observation point | 0 | 1 | 0 | 0 | 0 |
| Total | 5825 | 1023 | 511 | 11 | 39 |

Table S4 Number of positive populations for each detected avian coronavirus species

| Population | Number of positive populations | Number of positive populations for detected different species of avian coronaviruses | | | |
| --- | --- | --- | --- | --- | --- |
|  |  | Four species | Three species | Two species | One species |
| Live poultry markets | 289 | 2 | 30 | 107 | 150 |
| Wholesale markets | 140 | 1 | 22 | 49 | 68 |
| Poultry farmers | 51 | 0 | 0 | 1 | 50 |
| Slaughterhous | 27 | 0 | 0 | 7 | 20 |
| Backyard flocks | 4 | 0 | 0 | 0 | 4 |
| Wild bird observation point | 1 | 0 | 0 | 0 | 1 |

Fig S1 Phylogenetic tree constructed from the IBV positive samples based on the S1 gene sequence
